# Peripartum Phenomenon in Crioula Lanada Sheep Susceptible and Resistant to Gastrointestinal Nematodes

**DOI:** 10.3389/fvets.2020.00598

**Published:** 2020-09-08

**Authors:** Fabiellen Cristina Pereira, Cibele Longo, Caliê Castilho, Denise Pereira Leme, Jaqueline Seugling, César Cristiano Bassetto, Alessandro F. T. Amarante, Patrizia Ana Bricarello

**Affiliations:** ^1^Department of Animal Science and Rural Development, Agricultural Sciences Center, Federal University of Santa Catarina, Florianópolis, Brazil; ^2^Pro-Rectory Graduate Studies and Research, University of Oeste Paulista, São Paulo, Brazil; ^3^Institute of Biosciences, São Paulo State University (UNESP), Botucatu, Brazil

**Keywords:** sheep-nematoda, immune response, parasitology, blood parameters, breed, ewes

## Abstract

This study aimed at evaluating parasitological and blood variables in native breed Crioula Lanada sheep belonging to the same herd, to identify and compare susceptible or resistant individuals to gastrointestinal nematodes during gestation and lactation phases. For this purpose, 18 Crioula sheep were used within 2 years of evaluation, in which blood and feces samples and weight of the animals were taken from their 4th month of gestation until the weaning of their lambs, in the 4th month postpartum. Feces samples were used for counting eggs per gram of feces (EPG) and, thus, to identify 12 resistant ewes (EPG < 1,000) and 6 susceptible (EPG > 1,000) to gastrointestinal nematodes. The identification of infective larvae was also performed. Blood was used for analysis of packed cell volume (PCV), eosinophil count, total plasma protein, and immunoglobulin G level against *Haemonchus contortus* infective larvae. The Kruskal–Wallis non-parametric comparison test was used to evaluate the differences between days of parturition and linear mixed-effects model using package lme4 in R to evaluate the groups. The main parasite species observed in the feces samples were *Haemonchus, Trichostrongylus, Oesophagostomum, Ostertagia*, and *Cooperia* in similar proportions in both groups. Susceptible ewes presented peaks of EPG at the beginning of lactation and lower PCV values throughout the study. No difference between groups was observed concerning other blood variables or body weight, but some changes were observed along with the gestation and lactation phases within each group. The physiological response of sheep to nematode infection is a useful tool to identify the most susceptible individuals within the same breed and herd and to select the most genetically resistant individuals.

## Introduction

The success of sheep farming depends not only on the performance of the animal but also on its ability to adapt to the environment in which it is raised. The combination of these factors will determine whether it will be able to maintain its productivity, even during unfavorable situations, such as a supply of low-quality forage and diseases challenge. A major challenge in sheep farming is gastrointestinal parasitism, which causes large production losses (1), especially in more sensitive periods, such as the peripartum, in which sheep usually go through a physiological decrease in the immunological response, becoming even more susceptible to helminth infections (2, 3). As a consequence, an increased worm burden in the individual, increase of eggs excreted per gram of feces (EPG), and reduction of effector cells in the mucous membranes were observed (4). Concomitantly, there is an alteration in the packed cell volume (PCV) in the eosinophils count and antibodies: IgA, IgG, IgM, and IgE (1).

Anthelmintics have been extensively used in the prophylaxis of the parasitism. However, its efficacy has been diminished due to the occurrence of parasitic resistance in herds (5). Therefore, alternatives to chemical control have really been sought because of anthelmintic resistance and especially the potential for transfer of sub-effective doses to lambs in milk during lactation (6), among them, the ability of animals to generate an effective immune response. The ability for sheep to acquire and to express immunity against gastrointestinal nematodes is genetically controlled, and this varies substantially among different breeds (7).

The evaluation of the resistance status of a herd and the identification of more resistant animals or breeds are of major importance in genetic selection. The peripartum phenomenon is less pronounced in resistant ewes when compared to those of susceptible breeds (3, 8–11). More resistant breeds tend to have lower EPG counts, higher total plasma protein (TPP), and higher PCV, compared to those with the highest susceptibility (12). The most efficient immune mechanisms affect the parasites, preventing larvae establishment and development and decreasing the fecundity of adult worms (13), thus reducing egg excretion and contamination of pasture (14). Studies on lambs carried out in Brazil have demonstrated that native breeds, such as Crioula Lanada, tend to present high resistance to *Haemonchus contortus* infection (10, 15), an extremely important parasite in Brazil. Crioula Lanada sheep were brought from the Iberian Peninsula by colonizers. This wool breed is found in southern Brazil, but also in almost all South America countries, from Peru to Uruguay, which indicates a common origin dating back to the colonization of America. Crioula Lanada provably descends from Spanish Churra. Crioula sheep have some definitive advantages, such as precocity, higher fertility and resistance to endoparasites. The wool is naturally colored, varying from white to numerous shades of gray and beige to black, and is frequently used in handcrafts. The skins are often used over saddles and the meat is very much appreciated for its tenderness. The low population count is due to its replacement by specialized meat and wool breeds and to indiscriminate crossbreeding with other breeds (16).

Fecal examination has been the most important tool to diagnose parasitic gastroenteritis in ruminants and to verify the efficacy of anthelmintic treatments since there is an association between fecal egg counts and worm burden. However, due to the dynamic relationship between the parasite–host and the body immune response, which affects the outcomes of parasitic disease, the immunological status of the animals should be considered when interpreting the results of the fecal examination (17).

The objective of this study was to evaluate parasitological and blood variables in native breed Crioula Lanada sheep belonging to the same herd in Santa Catarina State, Brazil, to identify and compare individuals susceptible and resistant to gastrointestinal nematodes during the gestation and lactation phases.

## Materials and Methods

### Experimental Area

This experiment was undertaken at the Research and Extension Center in Agroecology belonging to the Center of Agricultural Sciences, Federal University of Santa Catarina (UFSC), Florianópolis, SC, Brazil. The animals are routinely maintained on a 5-hectare pasture platform divided into 47 paddocks. The climate in this region is characterized as Cfa–subtropical humid, according to the Köeppen climatic classification, with an average temperature of 20.1°C and an average annual rainfall of 1,462 mm (18). The laboratory analyses were carried out at the Laboratory of Animal Parasitology at the Center of Agricultural Sciences at UFSC and the Laboratory of Helminthology at the Department of Parasitology of the Institute of Biosciences of UNESP, Botucatu, SP, Brazil.

### Animals and Data Collection

The project was approved by the Ethics Committee on Animal Use of the Federal University of Santa Catarina (CEUA/UFSC) under the protocol N°PP00929. In the 1st year, 20 ewes of the Crioula Lanada breed were acquired already pregnant, mated without estrus synchronization with rams of the same breed. Most of them (18/20) lambed in autumn and winter. In the 2nd year, the same 20 sheep were synchronized by hormonal protocol and inseminated at fixed time (FTAI), resulting in 80% of pregnancies (16/20) and parturition peak in winter. The semen used originated from four sheep from the Conservation Center of Embrapa Pecuária Sul, Bagé, RS maintained by Embrapa Genetic Resources and Biotechnology, Brasília, DF and donated to UFSC.

For 2 years, throughout the study, these sheep were kept in a pasture under Voisin's Rational Grazing system with water and mineral salt *ad libitum*, including gestation and lactation periods. Grassland was naturally contaminated with free living stages of helminths. During the experiment, the sheep were treated with albendazole (10 mg/kg, Ibazole®, Ibasa), associated with levamisole hydrochloride (10 mg/kg, Ripercol® L 150 F, Fort Dodge) only when the EPG proved above 4,000 eggs and/or PCV equal to or <20%, to prevent possible deaths (19).

Blood and feces collections were performed fortnightly from the 4th month of gestation until the 4th month postpartum, when lambs were weaned, and weekly from the last month prior to parturition until the first month postpartum, respecting the gestation period of each sheep. Weighing of the animals was performed in the same period, and it was completed in the third month postpartum. Feces samples were collected directly from the rectum and blood samples were collected by jugular puncture in sterile glass vials containing heparin.

### Laboratory Analyses

The definition of the following parasitological and blood parameters was based on studies performed by Amarante et al. (19) and Bassetto et al. (20) for EPG, PCV, TPP, and blood eosinophils and Bricarello et al. (21) for IgG.

#### Parasitology

Feces samples were used for nematode EPG, performed using modified Gordon and Whitlock (22) technique. Resistant (EPG < 1,000) and susceptible (EPG > 1,000) ewes were identified based on the individual means of EPG throughout the studied period. The definition of these values was based on studies carried out with Crioula Lanada sheep, adult females, or lambs, whose maximum peaks in EPG against natural infection were around 2,000 (10, 23). Larval cultures were performed according to Roberts and O'Sullivan (24) method. The infective larvae obtained were identified according to Keith (25).

#### Blood Parameters

From the collected blood, 5 ml was centrifuged for 20 min at 2,000 *g*, to obtain the plasma, which was stored at −20°C for later determination of immunoglobulin G (IgG) concentration. IgG circulating antibodies against total antigen (L3) of *H. contortus* infective larvae were measured applying ELISA technique (21). The production of antigens for third-stage infective larvae (L3) *H. contortus* was previously described by Amarante et al. (26).

Silva et al. (27) previously described this protocol. Some modifications were made for carrying out this test. The plates were coated with 2 μg of antigen per milliliter, diluted in carbonate buffer (pH 9.6). Each wash was performed three times, rotating through 180° and re-washing three times. The used negative control (NC) was a worm-free animal, previously described by Santos et al. (28). The used plasma positive control (PC) was from animal fed with hay, artificially infected with *H. contortus* and *Trichostrongylus colubriformis* every 3 days for 84 days, and finally the conjugate was diluted at 1:40,000. Results for IgG were expressed as the percentage of the optical density value of the positive reference serum.

The determination of the PCV was performed by Microhematocrit method and of the TPP by an ocular refractometer (Atago®). TPP analyses started within 90–60 days before parturition.

Blood eosinophil counting (EOS) was performed in a Neubauer chamber after staining with Carpentier solution (29) and expressed in number of cells per microliter of blood.

### Statistical Analysis

Out of the total 18 ewes used for the study in 2014, six were considered susceptible and 12 were considered resistant to gastrointestinal nematodes. In 2015, the total of the ewes used for the study was 16, the same 6 considered susceptible and 10 that were considered resistant. The two extra ewes from the resistant group in 2014 were not pregnant in 2015. Therefore, the final separation of the sheep in two groups considering both years of study accounted for *N* = 6 on the susceptible group and *N* = 12 on the resistant one.

Taking into account that the period of gestation differed within ewes and years, we defined the date of parturition as being day zero and a range of 150 days before (−150 to 0; BP) and 150 days after the parturition (0 to + 150; AP), as the period of observation of data. Both ranges of 150 days, either BP or AP, were then divided into five periods of days to categorize response classes (data point): 150–90, 90–60, 60–30, 30–15, and 15–0 BP; 1–15, 15–30, 30–60, 60–90, and 90–150 AP. The individual measurements of ewes were then distributed into these data points and grouped as mean values per group, considering the data taken on the 2 years of study.

The non-parametric Kruskal–Wallis comparison test was used to evaluate the differences among response classes from the days of observation before and after parturition, since there was no normality of residuals of models after data transformation and it was verified by the Shapiro–Wilk test (*P* < 0.05). The error probability of <0.05 was accepted as having a significant difference.

Mixed-effects linear models (*lmer* function of the *lme4* package) were fitted to assess the effect of groups, resistant and susceptible, twin pregnancy, and period of days (response classes) on each measured variable, considering animals and year of study as random effects. *P*-values were obtained by Wald χ^2^ test type II (*P* < 0.05 or *P* < 0.01).

The R (30) computer program was used for all the analyses.

## Results

In the first year, eight sheep had to be treated with anthelmintic; two of them had been identified as resistant but presented egg counts higher than 4,000 eggs between 15–0 BP and 1–15 AP. One of those two resistant ewes was pregnant with twins and was treated twice; the other was treated three times. Among the six remaining ewes that were identified as susceptible, one of them has been treated five times, one has been treated three times, and the remaining sheep has been treated twice. In the second year, only susceptible ewes had to be treated. In total, five were treated, in which four had already been treated in 2014. One sheep was medicated once, and the remaining ones were medicated twice.The treatments were mostly concentrated during the 5th month of gestation and onset of lactation. In 2015, six ewes were pregnant with twins, two from the resistant group, one being the same one that was pregnant with twins in 2014, and four belonging to the susceptible group; two of these four were treated with anthelmintic twice.

**Figure 1:**
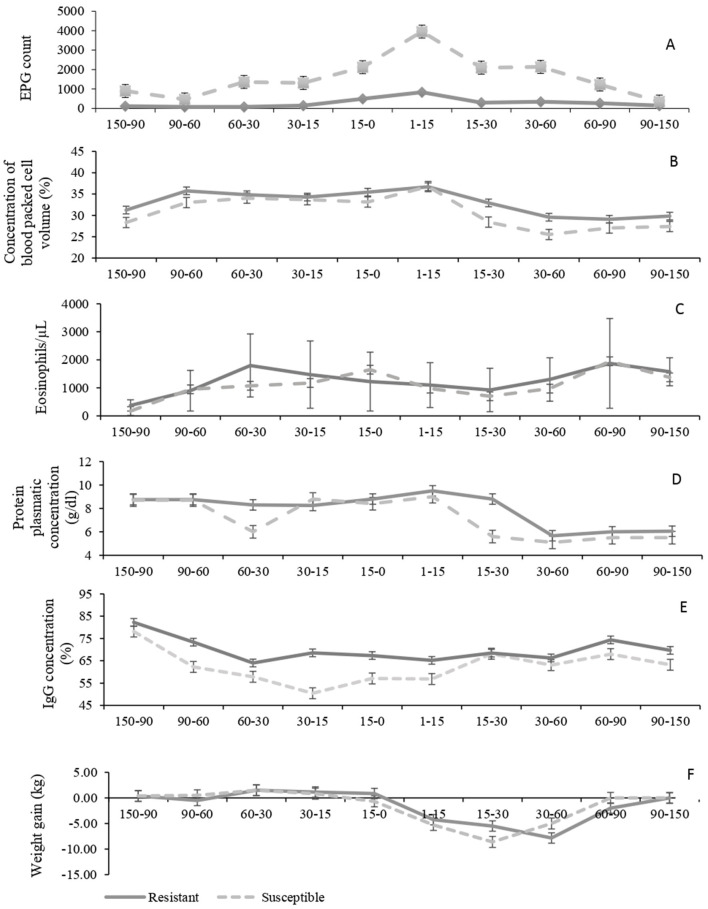
Mean values of egg counts per gram of feces (EPG) **(A)**, packed cell volmne (PCV) **(B)**, blood eosinophil counts **(C)**, total plasma protein (TPP) **(D)**, concentrations of IgG **(E)** and weekly weight variation **(F)** in Crioula Lanada ewes, susceptible (*N* = 6) and resistant (*N* = 12) to gastrointestinal nematodes during the gestation and lactation phases in the 2 years evaluated: 150–0 days before parturition and 1–150 days postpartum. Error bars indicate standard error of mean.

Variables analyzed comparing susceptible and resistant ewes during gestation and lactation periods are shown in Figure 1. There was an effect of group and period of days on EPG values (*P* < 0.001 for both), but there was no effect of twin pregnancy (*P* = 0.51). Resistant sheep presented a relatively stable mean lower than 1,000 (with individual values ranging from 0 to 8,600) throughout the assessed period. Susceptible ewes presented EPG peaks around 5,000 at the beginning of lactation (with individual values from 0 to 12,200) and received a greater number of anthelmintic treatments in this period. The coprocultures carried out in the first year showed the occurrence of parasites of the genus *Haemonchus, Trichostrongylus, Oesophagostomum*, and *Ostertagia*. In the second year, there was also the occurrence of *Cooperia* (Table 1). The richness of parasite genera was similar between susceptible and resistant ewes.

**Table 1:** Mean monthly values of nematode larvae obtained by the coproculture method with feces samples taken from ewes during gestation and lactation period in 2 years (1 and 2) of study.

	**Hae**^*******a*******^		**Tricho**^*******b*******^		**Oeso**^*******c*******^		**Ost**^*******d*******^		**Coop**^*******e*******^	
	**1**	**2**	**1**	**2**	**1**	**2**	**1**	**2**	**1**	**2**
April	81	25	19	16	0	25	0	0	0	34
May	80	13	14	10	0	22	6	2	0	53
June	80	16	16	5	0	1	4	1	0	77
July	60	17	30	6	0	8	9	1	0	68
August	64	37	16	5	1	6	19	5	0	45
September	66	51	20	3	6	3	8	2	0	40
October	70	28	11	30	11	9	8	0	0	33
November	81	40	10	12	1	9	8	0	0	39

a Haemonchus;

b Trichostrongylus;

c Oesophagostomum;

d Ostertagia;

e *Cooperia*.

For eosinophils, TPP, IgG, and body weekly weight gain, there was no group mean differences or twin pregnancy effects (*P* > 0.05), but changes within each group along with the advancement of the assessed phases (*P* < 0.01). Eosinophil values increased in both groups close to parturition, declined at the beginning of lactation, and reached a peak between the second and the third lactation months. There was a significant reduction in TPP and in weekly weight gain after birth (*P* < 0.001). Although there was no effect of group on weekly weight gain, it can be observed in Figure 1 that this reduction was numerically higher for the susceptible ewes. After the third month of lactation, values of weight gain returned to normal for both groups. Concentrations of IgG decreased through pregnancy (*P* < 0.001), but it starts to increase 15 days before birth along the lactation period. Values of PCV varied between groups (*P* = 0.04) and period of days (*P* < 0.001). In both groups, PCV values increased as soon as the birth approached, decreased after 15 days, and remained constant onwards. However, PCV values were lower for susceptible ewes than for resistant ewes throughout the study, except at data point 1–15 AP. There was a tendency of twin pregnancy effect for PCV values (*P* = 0.05).

## Discussion

This is the first study to evaluate the immune response of Crioula Lanada sheep naturally infected by gastrointestinal nematodes during gestation and lactation, comparing susceptible, and resistant females belonging to the same herd.

In general, Crioula Lanada sheep are considered rustic and resistant to helminth compared to Corriedale breed (10, 15). In this study, however, some susceptible individuals to nematode infections were identified. Although these are the minority of the herd, 33%, it is important to have them identified through parasitological and immunological parameters such as eosinophil counts and humoral response as well as molecular techniques, in order to select the most resistant breeders for offspring.

The considered susceptible ewes presented an average of 5000 EPG. High levels of EPG are not always an indicator of parasitic clinical manifestations. However, in stressful situations, like peripartum, infections can be exacerbated, resulting in clinical signs of parasitism, such as anemia, apathy, prostation, and weight loss (17). Peripartum is characterized by physiological depression of immunity and increase of EPG values at different intensities and moments. Peaks may occur early in lactation (31), or in the course of it (32), depending on the breed, but differences can be noticed even in ewes belonging to the same breed. Research carried out with Santa Inês hair sheep showed this variation. Sasa et al. (31) observed EPG peak at the time and intensity, similar to this study. However, Rocha et al. (9), reported the peak in the gestation phase, specifically in the fifth month for the Santa Inês sheep, and earlier (third month of gestation) and more pronounced peripartum for Ile de France sheep. This highlights the existence of physiological variations for animals belonging to the same breed and in the same herd in immune defense condition. In some cases, EPG increase may also occur in non-pregnant sheep in the lot, due to excessive contamination of the environment caused by the most susceptible sheep in the herd where they are kept (33).

Coprocultures demonstrated the occurrence of several genera of nematodes parasitizing the animals. Infection with *Cooperia* worms in the second year occurred after keeping adult cattle in the same grazing area where ewes were raised, in summer of the same year. This management was carried out due to the surplus forage in the paddocks. Based on measurements of *Cooperia* infective larvae of coprocultures, it is speculated that they are *C. puntata* and *C. pectinata* species, common parasite of cattle in tropical countries. Increased helminth diversity may be the result of lower anthelmintic treatments in the herd.

The susceptibility to diseases (infectious and metabolic—gestation toxemia) during gestation and lactation is the result of a decrease in the physiological defense capacity of the organism (34), since the host tends to prioritize the transmission of their genes and the survival of their offspring over its own immunity (35), allocating a considerable amount of nutrients for the reproductive functions (34). Some variables tend to reduce in the most susceptible animals as gestation and lactation advance, especially the TPP, which decreases approximately 0.0556 g/dl per day (36). This decrease is even more pronounced in pregnancy of twins (37); indeed, four of the susceptible ewes were pregnant with twins, which probably made them less resistant to parasitism, although no significant difference was found between ewes pregnant with one or twins. Twin-bearing ewes generally have higher egg counts and worm burden than their single breeding counterparts, and this takes place during both pre- and the postpartum period (38, 39). Twin-bearing ewes also have a better response to nutritional supplement than single-bearing ewes (38). Our susceptible ewes only had a peak of FEC close to the delivery period, and values were lower than susceptible ewes from other breeds (40, 41), meaning that Crioula breed is still more resistant. In this case, selecting individuals among different breeds may be favorable even for Crioulas pregnant with twins; besides, a combination of genetic selection and nutritional supplementation in Crioula herd would be effective management against parasitism. Contrary to what Ribeiro et al. (36) reported, we did not observe a constant regression on TPP but a marked decline after parturition (from 8.6 to 5.8 g/dl), observed in BlackBelly (32) and Ile de France sheep as well (8, 9). Values of TPP may reach around 5 g/dl in susceptible ewes (12) during the postpartum. In this study, however, these concentrations were also found in resistant ewes, emphasizing the physiological immunity decrease during peripartum. Offering a high-energy and high-protein diet in such period plays a vital role in minimizing infection damage and improving animal performance (42). Dietary supplementation aids replacing nutrients and maintaining the body mass during postpartum of sheep, making them more resistant and apt to cope with this sensitive period (2, 43). Taking into account that either susceptible and resistant ewes were submitted to the same management, it seems that the nutritional issue had greater influence than the animal's genetics over the plasma protein concentration since they had similar responses to this variable.

The slight increase observed in PCV during gestation is attributed to the greater need of oxygen transport in pregnant ewes, because of their higher metabolic rate compared to non-pregnant ewes (44). However, the EPG peak at the parturition leads to a reduction in the PCV during lactation, in response to the susceptibility of the females to the infection especially when the parasite has the behavior of feeding from the host blood, such as *H. contortus* (32). The observed decrease was considered critical only in the group of susceptible ewes, reaching 25% probably because most ewes from this group were pregnant with twins. Resistant ewes responded well, maintaining levels not lower than 29%. According to Zaros et al. (12), levels of up to 27% are reasonable; however, PCV around 20% and TPP levels around 5.3 g/dl begin to reflect the clinical signs of nematode infection: anemia, hypoproteinemia, apathy, and inappetence.

In relation to eosinophil, the counts varied throughout the period, but with values in general high in both groups (1,255 ± 875 μl^−1^ resistant vs. 1,100 ± 745 μl^−1^ susceptible), presenting marked eosinophilia. The eosinophilia indicates a higher activity of these cells in the defense of the organism against the parasites (20), either protecting it against the development of larvae or acting on their expulsion (10). Thus, eosinophilia is expected during a parasitic infection (13), especially when there is a peak of EPG. The blood eosinophils migrate to the damaged tissue during the infection, releasing granulated secondary proteins for healing (45). Its action is for regulating immunity and parasitic resistance, not being associated solely with resistant breeds (46). Therefore, its higher concentration may indicate a lack of the previous contact with the parasite or an increase in resistance to it (47). The same occurs with IgG serum levels. In a first contact with the parasite, the initial response may be low, followed by a gradual increase (27). In contrast, the high antigen-induced response in sheep may be due to sheep's low susceptibility or to a reinfection scenario (48). However, despite the importance of antibody production in triggering reactions and stimuli in response to an infection, immunological defense mechanisms in sheep are still not entirely clear.

Levels of IgG against *H. contortus* larvae declined as parturition approached, which may indicate insufficient prior contact with the parasite (28) or weakness in the organism defense. In contrast, a high level of IgG not only represents the occurrence of infection (27) but also may suggest a better immune response of the organism (49). Usually, the level of IgG occurs inversely to the EPG, since antibodies are directly responsible for preventing the establishment of larvae in the organism and indirectly responsible for its elimination (49). However, IgG values are not the most effective parameters to be considered for genetic selection (50).

The reduction in the number of anthelmintic treatments for sheep should also be considered when interpreting other parameters. Treatments occurred mostly during lactation and mainly in ewes that were pregnant with twins, while only two resistant ewes were medicated. The immunological status of sheep contributes to the identification of susceptible and resistant individuals to gastrointesinal nematodes (17). Resistance is an inherited characteristic by the offspring, and an individual's genetics can contemplate their resistance or their susceptibility to different stages of challenge; therefore, genetic selection, even among individuals of the same breed, is an efficient strategy for herd improvement (51).

In addition to the major problem of anthelmintic resistance in livestock around the world, the environmental risks of the excretion of these substances are completely neglected by competent authorities in South America and can have disastrous consequences, leading to contamination of soil, water, pastures, and food. Most anthelmintic are classified as of possible high impact, depending on how they are applied. The period of maximum excretion of residues is more transient in sheep manure than cattle's, but the excretion of reduced levels may continue for longer, extending the drug sublethal effects into the environment (52).

## Conclusion

Crioula Lanada sheep belonging to the same herd in Santa Catarina presented a variable immune response to gastrointestinal nematodes during gestation and lactation phases. Parasitological and blood variables contributed to identify individuals with higher resistance to the parasitism. The use of resistant breeds and/or individuals in sheep breeding programs is intended to keep herds to a satisfactory degree of resistance to gastrointestinal nematode infections, thus improving sheep farming with less often a prescription for antiparasitic treatments.

## Data Availability Statement

The datasets generated for this study are available on request to the corresponding author.

## Ethics Statement

The animal study was reviewed and approved by CEUA UFSC.

## Author Contributions

PB was responsible for project administration and guided and conducted all research. PB, DL, and CC originally formulated the idea and developed methodology. FP and JS conducted fieldwork and laboratory work. CL developed the mathematical models and performed statistical analyses. CB and AA conducted immunological laboratory tests. FP, PB, CL, CC, and DL analyzed the data and wrote the manuscript. All authors commented on manuscript drafts and gave the final approval for publication.

## Conflict of Interest

The authors declare that the research was conducted in the absence of any commercial or financial relationships that could be construed as a potential conflict of interest.

## References

[1] Bishop SC Possibilities to breed for resistance to nematode parasite infections in small ruminants in tropical production systems. Animal. (2012) 741–7. 10.1017/S1751731111000681 22558922

[2] Bassetto CC Almeida FA Newlands GFJ Smith WD Castilhos AM Fernandes S Trials with the Haemonchus vaccine, Barbervax®, in ewes and lambs in a tropical environment: nutrient supplementation improves protection in periparturient ewes. Vet Parasitol. (2018) 264:52–7. 10.1016/j.vetpar.2018.11.006 30503092

[3] Amarante AFT Craig TM Ramsey WS Sayed NME Desouki AY Bazer FW Comparison of naturally acquired parasite burdens among Florida native, rambouillet and crossbred ewes. Vet Parasitol. (1999) 85:61–9. 10.1016/S0304-4017(99)00103-X 10447193

[4] Cardoso EC Oliveira DR Dourado AP Araújo CV Ortalani EL Brandão FZ Peso e condição corporal, contagem de OPG e perfil metabólico sanguíneo de ovelhas da raça santa inês no periparto, criadas na região da baixada litorânea do estado do rio de janeiro. Rev Brasil Cien Vet. (2010) 17:77–82. 10.4322/rbcv.2014.148

[5] Oliveira PA Riet-Correa B Estima-Silva P Coelho ACB Santos BL Costa MAP Multiple anthelmintic resistance in Southern Brazil sheep flocks. Rev Bras Parasitol Vet. (2017) 26:427–32. 10.1590/s1984-29612017058 29069158

[6] Leathwick DM Miller CM Fraser K Selection for anthelmintic resistant *Teladorsagia circumcincta* in pre-weaned lambs by treating their dams with long-acting moxidectin injection. Int J Parasitol Drugs Drug Res. (2015) 5:209–14. 10.1016/j.ijpddr.2015.11.001 PMC4847000 27120068

[7] Stear MJ Murray M Genetic resistance to parasitic disease: particularly of resistance in ruminants to gastrointestinal nematodes. Vet Parasitol. (1994) 54:161–76. 10.1016/0304-4017(94)90089-2 7846849

[8] Rocha RA Bricarello PA Silva MB Houdijk JG Almeida FA Cardia DF Influence of protein supplementation during late pregnancy and lactation on the resistance of santa ines and ile de France ewes to haemonchus contortus. Vet Parasitol. (2011) 181:229–38. 10.1016/j.vetpar.2011.03.055 21726941

[9] Rocha RA Amarante AFT Bricarello PA Comparison of the susceptibility of santa inês and ile de France ewes to nematode parasitism around parturition and during lactation. Small Rumin Res. (2004) 55:65–75. 10.1016/j.smallrumres.2003.12.004

[10] Bricarello PA Gennari SM Oliveira-Sequeira TCG Vaz CMSL Gonçalvez IG Echevarria FAM Worm burden and immunological responses in corriedale and crioula lanada sheep following natural infection with *Haemonchus contortus*. Small Rumin Res. (2004) 51:75–83. 10.1016/S0921-4488(03)00188-3

[11] Wanyangu SW Mugambi JM Bain RK Duncan JL Murray M Stear MJ Response to artificial and subsequent natural infection with *Haemonchus contortus* in red massai and dorper ewes. Vet Parasitol. (1997) 69:275–82. 10.1016/S0304-4017(96)01129-6 9195737

[12] Zaros LG Neves MRM Benvenuti CL Navarro AMC Sider LH Coutinho LL Response of resistant and susceptible Brazilian Somalis crossbreed sheep naturally infected by *Haemonchus contortus*. Parasitol Res. (2014) 113:1155–61. 10.1007/s00436-014-3753-8 24425452

[13] Ahmed AM Sebastiano SR Sweeney T Hanrahan JP Glynn A Keane OM Breed differences in humoral and cellular responses of lambs to experimental infection with the gastrointestinal nematode *Teladorsagia circumcincta*. Vet Res. (2015) 46:1–9. 10.1186/s13567-014-0137-0 25827901 PMC4329660

[14] Greer AW Trade-offs and benefits: implications of promoting a strong immunity to gastrointestinal parasites in sheep. Parasite Immunol. (2008) 30:123–32. 10.1111/j.1365-3024.2008.00998.x 18186772

[15] Bricarello PA Gennari SM Oliveira-Sequeira TCG Vaz CMSL Gonçalvez IG Echevarria FAM Response of corriedale and crioula lanada sheep to artificial primary infection with *Haemonchus contortus*. Vet Res Commun. (2002) 26:447–57. 10.1023/a:1020538424876 12241098

[16] Mariante AS Cavalcante N Animals of the Discovery. Domestic Breeds in the History of Brazil. Brasília: Embrapa Sede/Embrapa Recursos Genéticos e Biotecnologia (2000). p. 232.

[17] Amarante AFT Amarante MRV Advances in the diagnosis of the gastrointestinal nematode infections in ruminants. Braz J Vet Res Anim Sci. (2016) 53:127–37. 10.11606/issn.1678-4456.v53i2p127-137

[18] Alvares CA Stape JL Sentelhas PC De Moraes G Leonardo J Sparovek G Köppen's climate classification map for Brazil. Meteorol Zeitschrift. (2013) 22:711–28. 10.1127/0941-2948/2013/0507

[19] Amarante AFT Bricarello PA Rocha RA Gennari SM Resistance of Santa Ines, Suffolk and Ile de France lambs to naturally acquired gastrointestinal nematode infections. Vet Parasitol. (2004) 120:91–106. 10.1016/j.vetpar.2003.12.004 15019147

[20] Bassetto CC Silva BF Fernandes S Amarante AFT Contaminação da pastagem com larvas infectantes de nematoides gastrintestinais após o pastejo de ovelhas resistentes ou susceptíveis à verminose. Rev Brasil Parasitol Vet. (2009) 18:63–8. 10.4322/rbpv.01804012 20040212

[21] Bricarello PA Zaros LG Coutinho LL Rocha RA Silva MB Kooyman FNJ Immunological response and citokine gene expression analysis to *Cooperia punctata* infections in resistant and susceptible nelore cattle. Vet Parasitol. (2008) 155:95–103. 10.1016/j.vetpar.2008.03.016 18513872

[22] Gordon HM Whitlock HV A new technique for counting nematode eggs in sheep faeces. J Counc Sci Ind Res. (1939) 12:50–2.

[23] Romero-Escobedo E Torrez-Hernandes G Becerril-Pérez CM Alarcón-Zúñiga B Apodaca-Sarabia A Días-Rivera P A comparison of criollo and suffolk ewes for resistance to *Haemonchus contortus* during the periparturient period. J Appl Anim Res. (2018) 2119:16–23. 10.1080/09712119.2016.1252378

[24] Roberts FHS O'Sullivan PJ Methods for egg counts and larval cultures for strongyles infesting the gastrointestinal tract of cattle. Aust J Agric Res. (1950) 1:99–102. 10.1071/AR9500099

[25] Keith RK The differentiation of the infective larvae of some common nematode parasites of cattle. Aust J Zool. (1953) 1:223–35. 10.1071/ZO9530223

[26] Amarante AFT Susin I Rocha RA Silva MB Mendes CQ Pires AV Resistance of santa ines and crossbred ewes to naturally acquired gastrointestinal nematode infections. Vet Parasitol. (2009) 165:273–80. 10.1016/j.vetpar.2009.07.009 19656629

[27] Silva BF Bassetto CC Amarante AFT Immune responses in sheep naturally infected with Oestrus ovis (Diptera: Oestridae) and gastrointestinal nematodes. Vet Parasitol. (2012) 190:120–6. 10.1016/j.vetpar.2012.06.004 22770703

[28] Santos MC Xavier JK Amarante MRV Bassetto CC Amarante AFT Immune response to *Haemonchus contortus* and *Haemonchus placei* in sheep and its role on parasite specificity. Vet Parasitol. (2014) 203:127–38. 10.1016/j.vetpar.2014.02.048 24670867

[29] Dawkins HJS Windon RG Eagleson GK Eosinophil responses in sheep selected for high and low responsiveness to *T. colubriformis*. Int J Parasitol. (1989) 19:199–205. 10.1016/0020-7519(89)90008-8 2722393

[30] R Core Team R: A. Language and Environment for Statistical Computing. Vienna: R Foundation for Statistical Computing (2018).

[31] Sasa A Neves EP Castilho MFO Mexia AA Infecção helmíntica em ovelhas santa inês no periparto criadas na região do pantanal brasileiro. Rev Brasil Saúde Prod Ani. (2008) 9:321–6.

[32] Garduño RG Arellano MEL Felipe MMC Gives PM Marcelino LA Díaz GJ Immune and haematological parameters of blackbelly ewes. Rev Colomb Cien Pecuar. (2016) 30:219–30. 10.17533/udea.rccp.v30n3a05

[33] Pinto JM Relação entre o periparto e a eliminação de ovos de nematóides gastrintestinais em cabras anglo-nubiana naturalmente infectadas em sistema semi-extensivo de produção. Rev Bras Parasitol Vet. (2008) 17:138–43. 20059833

[34] Saccareau M Moreno CR Kyriazakis I Faivre R Bishop SC Modelling gastrointestinal parasitism infection in a sheep flock over two reproductive seasons : *in silico* exploration and sensitivity analysis. Parasitology. (2016) 2016:1509–31. 10.1017/S0031182016000871 27356626

[35] Hoste H Torres-Acosta JFJ Quijada J Chan-Pérez I Dakheel MM Kommuru DS Interactions between nutrition and infections with *Haemonchus contortus* and related gastrointestinal nematodes in small ruminants. In: Gasser RB, von Samson-Himmelstjerna G, editors. Haemonchus contortus and Haemonchosis-Past, Present and Future Trends Adv Parasitol. (2016). p. 239–351. 10.1016/bs.apar.2016.02.025 27238007

[36] Ribeiro LAO Mattos RC Gonzalez FHD Wald VB Silva MA Rosa VL Perfil metabólico de ovelhas Border Leicester x Texel durante a gestação e a lactação. Rev Port Ciên Vet. (2004) 99:155–159.

[37] Balikci E Yildiz A Gurdogan F Blood metabolite concentrations during pregnancy and postpartum in akkaraman ewes. Small Rumin Res. (2007) 67:247–51. 10.1016/j.smallrumres.2005.10.011

[38] Kahn LP Knox MR Walkden-Brown SW Lea JM Regulation of the resistance to nematode parasites of single-and twin-bearing merino ewes through nutrition and genetic selection. Vet Parasitol. (2003) 114:15–31. 10.1016/S0304-4017(03)00099-2 12732463

[39] Houdijk JGM Jessop NS Kyriazakis I Nutrient partitioning between reproductive and immune functions in animals. Proc Nutr Soc. (2001) 60:515–25. 10.1079/PNS2001114 12069405

[40] Gasparina JM Fonseca L Loddi MM Martins ADS Rocha RAD Resistance of ewes to gastrointestinal nematode infections during the peripartum and dry periods and the performance of their lambs. Rev Brasil de Saúde e Prod Animal. (2019) 20:2019 10.1590/s1519-9940200282019

[41] Kahn LP Knox MR Gray GD Corbett JL Enhancing immunity to nematode parasites in pregnant and lactating sheep through nutrition and genetic selection. Recent Adv Anim Nutr Aus. (1999) 12:15–22.

[42] David CMG Costa RLD Parren GAE Rua MAS Nordi ECP Okamoto F Sugarcane and mulberry silage supplementation of sheep during the peripartum period. Trop Anim Health Prod. (2015) 47:765–72. 10.1007/s11250-015-0791-x 25761641

[43] Sakkas P Houdijk JG Athanasiadou S Kyriazakis I Sensitivity of periparturient breakdown of immunity to parasites to dietary protein source. J Anim Sci. (2012) 90:3954–62. 10.2527/jas.2011-4829 22665670

[44] El-Sherif MMA Fawzia A Changes in some blood constituents of Barki eyes during pregnancy and lactation under semi-arid conditions. Small Rumin Res. (2001) 40:269–77. 10.1016/S0921-4488(01)00174-2 11323212

[45] Mcrae KM Stear MJ Good B Keane OM The host immune response to gastrointestinal nematode infection in sheep. Parasite Immun. (2015) 37:605–13. 10.1111/pim.12290 PMC4744952 26480845

[46] Bowdridge SA Zajac AM Notter DR St. Croix sheep produce a rapid and greater cellular of *Haemonchus contortus. Vet. Parasitol* (2015) 208:204–210. 10.1016/j.vetpar.2015.01.019 25698414

[47] Shakya KP Miller JE Lomaz LG Burnett DD Evaluation of immune response to artificial infections of *Haemonchus contortus* in gulf coast native compared with suffolk lambs. Vet. Parasitol. (2011) 181:239–47. 10.1016/j.vetpar.2011.03.051 21570191

[48] Vijayasarathi MK Sheeba A Sreekumar C Dhama K Immune responses to *Haemonchus Contortus* in sheep. J Immunol Immunopat. (2015) 17:79–85. 10.5958/0973-9149.2015.00011.8

[49] Zacharias F Guimarães JE Araújo RR Almeida MAO Ayres MCC Bavia ME Effect of homeopathic medicines on helminth parasitism and resistance of *Haemonchus contortus* infected sheep. Homeopathy. (2008) 97:145–51. 10.1016/j.homp.2008.05.004 18657774

[50] Gauly M Kraus M Vervelde L Van Leeuwen MAW Erhardt G Estimating genetic differences in natural resistance in rhön and merinoland sheep following experimental *Haemonchus contortus* infection. Vet Parasitol. (2002) 106:55–67. 10.1016/S0304-4017(02)00028-6 11992711

[51] Amarante AT Sustainable worm control practices in South America. Small Rumin Res. (2014) 118:56–62. 10.1016/j.smallrumres.2013.12.016

[52] Beynon SA Potential environmental consequences of administration of anthelmintics to sheep. Vet Parasitol. (2012) 189:113–24. 10.1016/j.vetpar.2012.03.040 22538093

